# Hyperreactivity to weak acoustic stimuli and prolonged acoustic startle latency in children with autism spectrum disorders

**DOI:** 10.1186/2040-2392-5-23

**Published:** 2014-03-12

**Authors:** Hidetoshi Takahashi, Takayuki Nakahachi, Sahoko Komatsu, Kazuo Ogino, Yukako Iida, Yoko Kamio

**Affiliations:** 1 Department of Child and Adolescent Mental Health, National Institute of Mental Health, National Center of Neurology and Psychiatry, 4-1-1 Ogawahigashicho, Kodaira, Tokyo 1878553, Japan

**Keywords:** Autism spectrum disorders, Acoustic startle response, Startle latency, Hyperreactivity, Quantitative autistic traits, Asian

## Abstract

**Background:**

People with autism spectrum disorders (ASD) are known to have enhanced auditory perception, however, acoustic startle response to weak stimuli has not been well documented in this population. The objectives of this study are to evaluate the basic profile of acoustic startle response, including peak startle latency and startle magnitude to weaker stimuli, in children with ASD and typical development (TD), and to evaluate their relationship to ASD characteristics.

**Methods:**

We investigated acoustic startle response with weak and strong acoustic stimuli in 12 children with ASD and 28 children with TD, analyzing the relationship between startle measures and quantitative autistic traits assessed with the Social Responsiveness Scale (SRS). The electromyographic activity of the left orbicularis oculi muscle to acoustic stimuli of 65 to 115 dB sound pressure level (SPL), in increments of 5 dB, was measured to evaluate acoustic startle response. The average eyeblink magnitude for each acoustic stimuli intensity and the average peak startle latency of acoustic startle response were evaluated.

**Results:**

The magnitude of the acoustic startle response to weak stimuli (85 dB or smaller) was greater in children with ASD. The peak startle latency was also prolonged in individuals with ASD. The average magnitude of the acoustic startle response for stimulus intensities greater than 85 dB was not significantly larger in the ASD group compared with the controls. Both greater startle magnitude in response to weak stimuli (particularly at 85 dB) and prolonged peak startle latency were significantly associated with total scores, as well as several subscales of the SRS in the whole sample. We also found a significant relationship between scores on the social cognition subscale of the SRS and the average magnitude of the acoustic startle response for stimulus intensities of 80 and 85 dB in the TD group.

**Conclusions:**

Children with ASD exhibited larger startle magnitude to weak stimuli and prolonged peak startle latency. These startle indices were related to several characteristics of ASD. A comprehensive investigation of acoustic startle response, including the magnitude of startle responses to weak stimuli and peak startle latency, might further our understanding of the neurophysiological impairments underlying ASD.

## Background

Sensory abnormalities have been considered a key feature of autism spectrum disorders (ASD) since the pioneering reports of Kanner [1]. Sensory abnormalities are frequently present in individuals with ASD, with a high prevalence of auditory, visual and tactile hyperreactivity, as well as hyporeactivity [2,3]. The ability to accurately process and interpret auditory information is often difficult for people with ASD (see O’Connor [4] for a review). Specifically, enhanced perception of simple, low-level stimuli is thought to contribute to the atypical auditory processing in this population (see Happé and Frith [5] and Mottron *et al*. [6] for reviews). Auditory hyperreactivity is the most common sensory-perceptual abnormality, with a prevalence ranging between 15% and 100% in people with ASD [2]. This abnormality is reported to interrupt behavioral adaptation [7], and sometimes even requires therapeutic intervention [8].

The acoustic startle reflex (ASR) is a commonly used neurophysiological measure for evaluating various aspects of information processing. Since ASR can be examined using similar nonlinguistic experimental paradigms across ethnic groups and species, it is considered to be one of the most promising neurophysiological measures for translational research.

Although several studies have investigated the response of people with ASD to weak acoustic stimuli, the startle response has not been thoroughly investigated for weak acoustic stimuli in this population. In humans, ASR is typically elicited by sound stimuli greater than 80 to 85 dB [9]. Most previous studies investigating ASR in ASD used startle stimuli of between 100 and 110 dB (typically 105 dB) [10-13], and could not find significant difference of ASR magnitude between ASD and controls. However, ASR to acoustic stimuli of 80 dB or less has not been well investigated in ASD. Khalfa *et al*. [14] reported that 63% of autistic individuals did not support stimulation above 80 dB. In addition, a brainstem audiometry study reported that 18% of the autism group presented normal auditory thresholds and auditory hyperreactivity with intolerance to click sounds above 70 dB [15]. Thus, ASR to weak acoustic stimuli around 70 to 80 dB appear to be atypical in ASD, and we hypothesize that the difference in startle magnitude between subjects with ASD and typical development (TD) might be larger for weak stimuli compared with strong stimuli.

The current study sought to investigate the basic ASR profile, including startle magnitude to acoustic stimuli ranging from weak to strong intensities and peak startle latency in children with ASD and TD. We also evaluated the relationship of startle measures to quantitative autistic traits assessed by the Social Responsiveness Scale (SRS) [16].

## Methods

### Participants

Sixteen Japanese children with ASD and 30 Japanese control children with TD (age 6 to 17 years) participated in this study. Subjects were recruited by local advertisements in Tokyo, Japan. Participants were diagnosed by an experienced child psychiatrist on the basis of current presentation and developmental history, as determined by medical record reviews and clinical interviews based on the Diagnostic and Statistical Manual of Mental Disorders, fourth edition, text revision (DSM-IV-TR) [17]. Diagnoses were confirmed using the Autism Diagnostic Interview-Revised (ADI-R) [18] and the Autism Diagnostic Observation Schedule (ADOS) [19]. The Wechsler Intelligence Scale for Children, third edition (WISC-III) [20] was used to estimate IQ. All the subjects exhibited an estimated IQ above 70 and were nonsmokers. None of the participants were currently receiving pharmaceutical treatment with psychotropic substances, with the exception of two boys with ASD, who were prescribed methylphenidate hydrochloride. Exclusion criteria included known hearing loss and central nervous system involvement other than autism. In addition, control subjects were excluded if they had any history of psychiatric diagnoses or learning disabilities.

Quantitative autistic traits of subjects were assessed by parents using the Japanese version [21] of the SRS [16]. The SRS items have been further categorized into five treatment subscales (social awareness, social cognition, social communication, social motivation and autistic mannerisms) [16]. Higher scores on the SRS indicate a higher degree of social impairment. Raw scores of SRS were converted to T-scores (with mean of 50 and standard deviation of 10) for gender.

The study procedure was conducted in accordance with the Declaration of Helsinki and approved by the Research Ethical Committee of the National Center of Neurology and Psychiatry, Tokyo, Japan. All subjects and their parents gave written informed consent after the study procedures had been fully explained to them.

### Startle response measurement

A commercial computerized human startle response monitoring system (Startle Eyeblink Reflex Analysis System Map1155SYS, NIHONSANTEKU Co., Osaka, Japan) was used to deliver acoustic startle stimuli, and record and score the corresponding electromyographic activity. The methods for stimulus presentation and eyeblink acquisition are described in detail elsewhere [22,23]. We also present the details of the startle response measurement in Additional file 1.

All auditory stimuli and background noise (broadband white noises of 1.346 Hz to 22.05 KHz) were delivered binaurally to subjects through stereophonic headphones. Startle eyeblink electromyographic responses were recorded from the left orbicularis oculi muscle. The eyeblink magnitude of every startle response was defined as the voltage of the peak electromyographic activity within a latency window of 20 to 120 ms following startle-eliciting stimulus onset. The data were stored and exported for analyses in microvolt values.

Participants were tested in a startle paradigm with a continuously presented 60 dB sound pressure level (SPL) background white noise. Acoustic stimuli consisted of broadband white noises lasting for 40 ms presented at intensities from 65 to 110 dB SPL, in 5 dB increments. Acoustic stimuli were presented six times at each intensity. All trials were presented in a fixed pseudorandom order, separated by intertrial intervals of 10 to 20 s (15 s on average). The startle paradigm consisted of a total of 60 trials. The session lasted approximately 20 min, including 5 min acclimation to the background noise.

The following startle measures were examined: 1) the average startle eyeblink magnitude in ASR to each pulse intensity (65, 70, 75, 80, 85, 90, 95, 100, 105 and 110 dB), designated as ASR65, ASR70, ASR75, ASR80, ASR85, ASR90, ASR95, ASR100, ASR105 and ASR110, respectively; and 2) the average peak startle latency, that is, the average peak startle latency of ASR among the trials which had an ASR larger than 60 microvolts.

Prior to data analyses, trials were discarded if the voltage of their peak electromyographic activity within a latency window of 0 to 20 ms following startle-eliciting stimulus onset was more than 60 microvolts. One girl with TD and one boy with ASD were unable to tolerate the startle stimuli and did not complete the session. Their data were excluded from the final analysis. One boy with TD and three children (one boy and two girls) with ASD were also excluded from further analyses because more than half of the trials at any stimulus intensity were discarded.

The demographic characteristics of the remaining subjects are presented in Table 1. The ASD and control groups did not differ significantly in terms of sex distribution. Age and estimated IQ did not differ significantly between groups. We did not find any significant gender differences for age or estimated IQ in each group. The T-scores in the SRS were significantly higher in the ASD group compared to controls (Table 1). We found no significant gender differences in terms of SRS scores between the groups. The subjects excluded from the startle response measurement analyses did not differ significantly from the included subjects in terms of SRS scores or demographic characteristics, such as age, sex distribution and estimated IQ. In addition, the number of discarded trials did not differ significantly between the ASD and TD groups (ASD 5.67 ± 5.97, TD 3.18 ± 5.06, U = 136.0, *P* = 0.338).

**Table 1:** Demographic data

**Characteristics**	**Typical development (TD)**		**Autism spectrum disorders (ASD)**				
	**(n = 28)**		**(n = 12)**				
					***χ***^**2**^	**df**	***P***
Male: female	15:13		9:3		1.607	1	0.205
	**Mean**	**SD**	**Mean**	**SD**	**U**		***P***
Age (months)	134.1	31.1	119.3	31.9	109		0.081
Estimated IQ	102.6	17.8	102.0	15.0	78.5		0.788
Social responsiveness scale T-score							
Total score	47.3	9.3	73.8	17.3	23		0.000**
Social awareness	45.4	10.9	65.6	12.0	25		0.000**
Social cognition	47.8	7.5	73.5	12.5	16.5		0.000**
Social communication	47.7	8.5	70.2	19.8	38.5		0.000**
Social motivation	45.2	9.4	57.8	17.1	87.5		0.017*
Autistic mannerisms	51.6	11.5	82.3	18.0	24.5		0.000**

ASD, Autism spectrum disorders; df, degrees of freedom; IQ, intelligence quotient; SD, standard deviation; TD, typical development. Mann–Whitney *U* test; *P <0.05; **P <0.01.

### Statistical analysis

We used *χ*^2^ tests (and Fisher’s exact test as appropriate) to compare categorical proportions. None of the startle measures, except peak startle latency (W = 0.961, *P* = 0.385), were found to be normally distributed based on the Shapiro–Wilk W statistic (*P* <0.05). None of SRS T-scores, except the SRS social awareness subscale (W = 0.967, *P* = 0.518) and the SRS social motivation subscale (W = 0.958, *P* = 0.327), were normally distributed. Therefore, we performed nonparametric analyses. The Mann–Whitney *U* test was used for comparison of mean SRS scores and startle measures. Spearman’s rank order correlations examined the relationship between startle measures and SRS scores. Stepwise multiple regressions were also performed with SRS scores acting as the dependent variable to evaluate the association between SRS scores and startle measurements and to confirm the results of the Spearman’s rank order correlations for reference. All *P* values reported here were two-tailed. Statistical significance was indicated by *P* values <0.05. Statistical analyses were performed using SPSS version 21 (SPSS Japan, Tokyo, Japan).

## Results

### Difference in startle measures between children with autism spectrum disorders (ASD) and typical development (TD)

**Figure 1:**
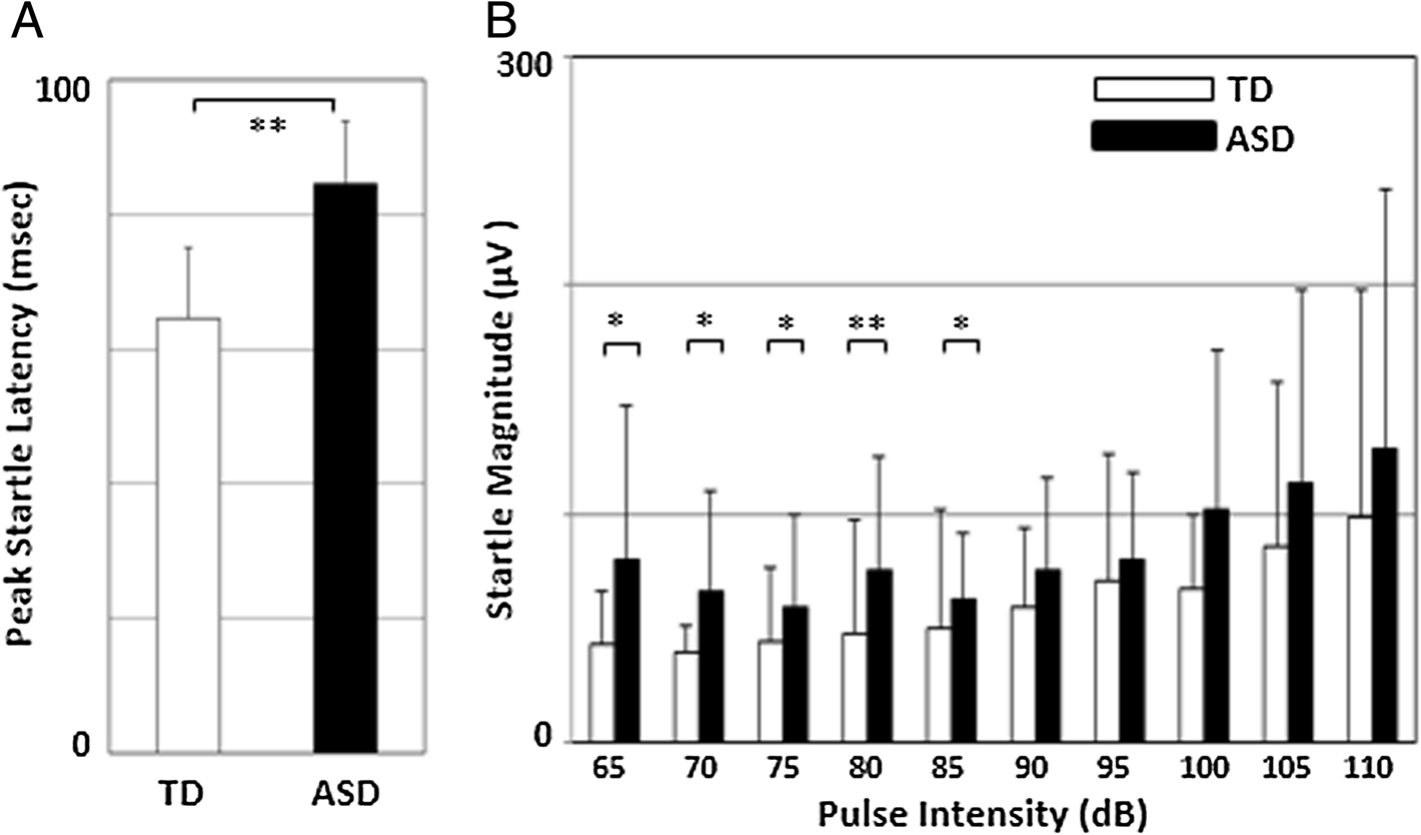
**Comparison of startle measures between children with autism spectrum disorders (ASD) and typical development (TD). (A)** Comparison of PSL between ASD and TD (ASD, n = 12; TD, n = 28). **(B)** Comparison of startle magnitude at each stimulus intensity between ASD and TD (ASD, n = 12; TD, n = 28). The figures show the means and the error bars show the standard deviation. Mann–Whitney *U* test; *P <0.05; **P <0.01. ASD, autism spectrum disorders; PSL, peak startle latency; TD, typical development.

Figure 1 shows the difference in startle measures between children with ASD and controls. Peak startle latency was significantly prolonged in children with ASD compared with controls (U = 22, *P* <0.001; Figure 1A). In addition, children with ASD exhibited significantly greater ASR magnitude in stimulus intensities at 65 to 85 dB (ASR65, U = 81, *P* = 0.010; ASR70, U = 95, *P* = 0.031; ASR75, U = 96, *P* = 0.034; ASR80, U = 53, *P* <0.001; ASR85, U = 80, *P* = 0.009; Figure 1B). Average ASR magnitude in stimulus intensities stronger than 85 dB were not significantly larger in the ASD group compared to controls.

### Relationship of startle measures to autistic traits

**Figure 2:**
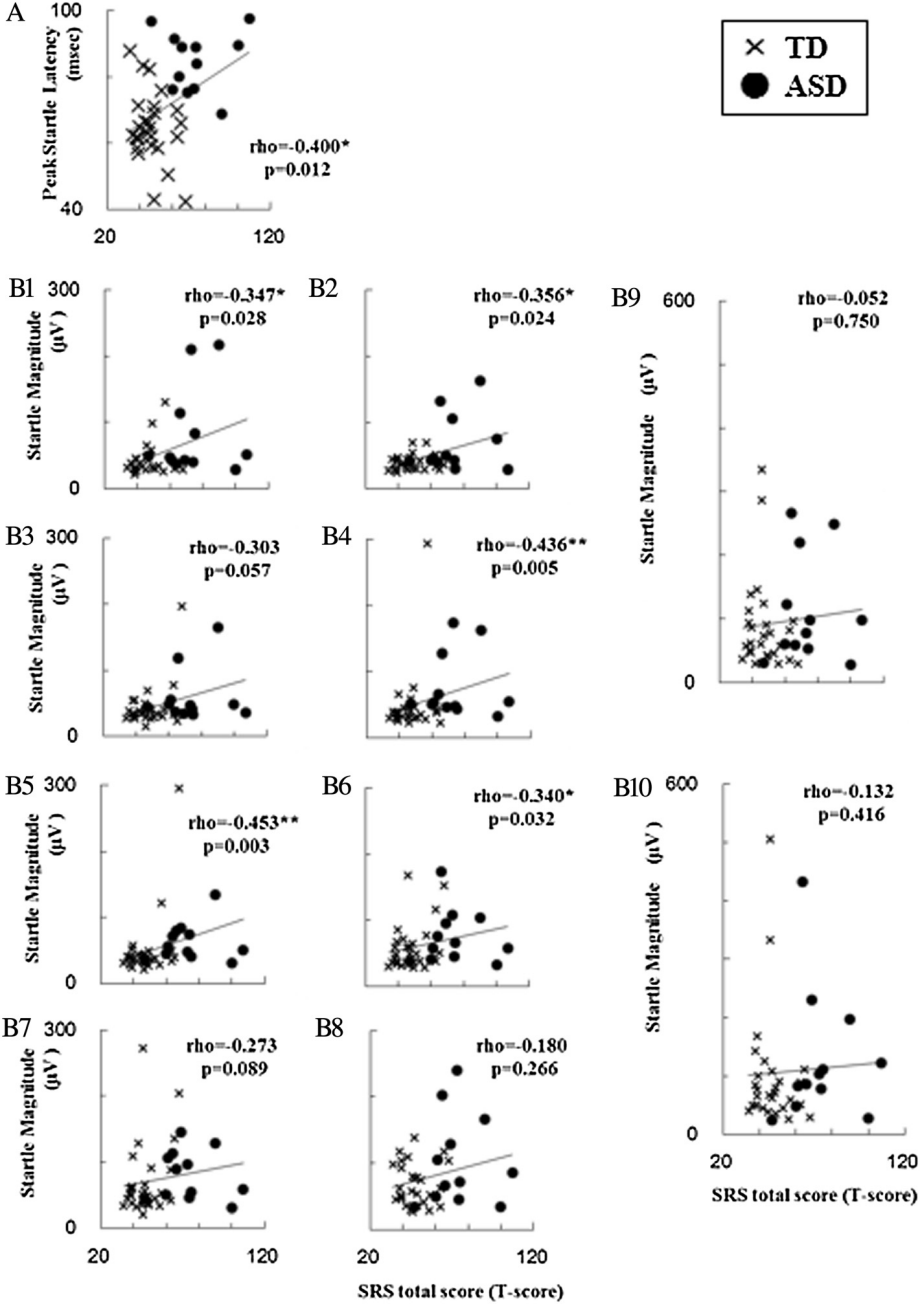
**Scatterplots of startle measures by T-score of Social Responsiveness Scale (SRS) total score. (A)** PSL for T-score of the SRS total score. **(B1)**, **(B2)**, **(B3)**, **(B4)**, **(B5)**, **(B6)**, **(B7)**, **(B8)**, **(B9)** and **(B10)** represent ASR65, ASR70, ASR75, ASR80, ASR85, ASR90, ASR95, ASR100, ASR105 and ASR110, respectively, for T-score of the SRS total score. Variables are rho. ASR65, ASR70, ASR75, ASR80, ASR85, ASR90, ASR95, ASR100, ASR105 and ASR110 are designated as the average startle eyeblink magnitude at stimulus intensities of 65, 70, 75, 80, 85, 90, 95, 100, 105 and 110 dB, respectively. n = 40 (ASD, n = 12; TD, n = 28). Spearman’s rank order correlation; *P <0.05; **P <0.01. ASD, autism spectrum disorders; ASR, acoustic startle reflex; PSL, peak startle latency of acoustic startle reflex; SRS, Social Responsiveness Scale; TD, typical development.

The relationship of startle measures to autistic traits evaluated with SRS is provided in Figure 2 and Table 2. As behavioral traits of ASD are suggested to present a continuous distribution across the population [24], we evaluated the relationships between startle measures and SRS scores within the whole group, including subjects with ASD and TD.

**Table 2:** Relationships between startle measures and T-scores on subscales of Social Responsiveness Scale (SRS)

**SRS scores**		**PSL**	**Startle response**									
			**ASR65**	**ASR70**	**ASR75**	**ASR80**	**ASR85**	**ASR90**	**ASR95**	**ASR100**	**ASR105**	**ASR110**
Social awareness	rho	0.38*	0.38*	0.28	0.34*	0.42**	0.38*	0.29	0.21	0.13	−0.03	−0.02
	*P*	0.016	0.015	0.083	0.031	0.007	0.015	0.071	0.183	0.437	0.855	0.910
Social cognition	rho	0.38*	0.29	0.37*	0.22	0.45**	0.50**	0.42**	0.38*	0.27	0.17	0.22
	*P*	0.018	0.072	0.020	0.167	0.004	0.001	0.008	0.016	0.095	0.290	0.180
Social communication	rho	0.30	0.28	0.30	0.30	0.38*	0.42**	0.35*	0.31	0.19	0.08	0.15
	*P*	0.064	0.081	0.057	0.059	0.015	0.008	0.025	0.055	0.243	0.627	0.355
Social motivation	rho	0.34*	0.27	0.18	0.13	0.13	0.10	0.05	−0.05	−0.06	0.06	0.14
	*P*	0.033	0.093	0.261	0.426	0.417	0.532	0.774	0.760	0.704	0.699	0.386
Autistic mannerisms	rho	0.42**	0.37*	0.41**	0.41**	0.48**	0.53**	0.31	0.21	0.10	−0.07	0.01
	*P*	0.008	0.019	0.008	0.009	0.002	0.000	0.051	0.197	0.522	0.677	0.936

ASR65, ASR70, ASR75, ASR80, ASR85, ASR90, ASR95, ASR100, ASR105 and ASR110 are designated as the average startle eyeblink magnitude at stimulus intensities of 65, 70, 75, 80, 85, 90, 95, 100, 105 and 110 dB, respectively. n = 40 (ASD, n = 12; TD, n = 28). Spearman’s rank order correlation; *P <0.05; **P <0.01. ASD, autism spectrum disorders; ASR, acoustic startle reflex; PSL, average peak startle latency of acoustic startle reflex; SRS, Social Responsiveness Scale; TD, typical development.

The T-score of SRS total score was significantly correlated with the peak startle latency as well as the average ASR magnitude of stimulus intensities at 65, 70, 80, 85 and 90 dB (Figure 1). Significant correlations of T-scores of several SRS subscales were also found in peak startle latency as well as average ASR magnitude of stimulus intensities at 90 dB or weaker (Table 2). Average ASR magnitude at stimulus intensities of 100 dB or greater did not show any correlations with SRS scores. We found a significant relationship between scores on the SRS social cognition subscale, ASR80 (rho = 0.392, *P* = 0.039) and ASR85 (rho = 0.398, *P* = 0.036) in the TD group. We did not find any other significant relationships between SRS scores and startle measures within each group.

We also conducted stepwise multiple regression analyses with T-score in the SRS as the dependent variable and the startle measures as independent variables. Prolonged peak startle latency and greater ASR85 were significantly associated with higher T-scores of SRS total score, as well as most of the subscales of the SRS (SRS total score, adjusted *R*^2^ = 0.34, F = 2, df = 10.59, *P* <0.001; peak startle latency (PSL), unstandardized regression coefficient (B) = 1.37, standard error (SE) = 0.34, standardized regression coefficient (beta) = 0.565, *P* <0.001; ASR85, B = 0.34, SE = 0.1, beta = 0.46, *P* = 0.002; social awareness, adjusted *R*^2^ = 0.31, F = 2, df = 9.56, *P* <0.001; PSL, B = 0.17, SE = 0.05, beta = 0.53, *P* <0.001; ASR85, B = 0.05, SE = 0.01, beta = 0.46, *P* = 0.002; social cognition, adjusted *R*^2^ = 0.38, F = 3, df = 8.67, *P* <0.001; PSL, B = 0.21, SE = 0.07, beta = 0.44, *P* = 0.003; ASR85, B = 0.05, SE = 0.02, beta = 0.35, *P* = 0.02; ASR70, B = 0.07, SE = 0.03, beta = 0.31, *P* = 0.031; social communication, adjusted *R*^2^ = 0.29, F = 2, df = 8.56, *P* = 0.001; PSL, B = 0.44, SE = 0.13, beta = 0.5, *P* = 0.002; ASR85, B = 0.12, SE = 0.04, beta = 0.46, *P* = 0.003; social motivation, adjusted *R*^2^ = 0.12, F = 1, df = 6.4, *P* = 0.016; PSL, B = 0.14, SE = 0.06, beta = 0.38, *P* = 0.016; autistic mannerisms, adjusted *R*^2^ = 0.38, F = 2, df = 12.53, *P* <0.001; PSL, B = 0.35, SE = 0.08, beta = 0.58, *P* <0.001; ASR85, B = 0.09, SE = 0.02, beta = 0.5, *P* <0.001).

## Discussion

In this study, we investigated ASR in children with ASD and TD using a startle paradigm with stimulus intensities ranging from weak (65 dB) to strong (110 dB) stimuli. Children with ASD exhibited significantly larger startle responses compared to TD controls to weak stimuli at 85 dB or smaller. The peak startle latency of ASR was significantly prolonged in children with ASD compared to controls. Prolonged startle latency and startle magnitude in response to weak stimuli, particularly at 85 dB, were associated with quantitative autistic traits assessed by SRS.

To the best of our knowledge, this is the first study to report an association between ASD and an increased startle response elicited by weak acoustic stimuli. Most previous studies investigating ASR in ASD employed acoustic stimuli of a single intensity, typically around 105 dB. The current finding that there was no significant group difference in ASR to stimuli stronger than 90 dB is consistent with these previous reports [10-13]. However, in our study, ASR to weak stimuli of 85 dB or weaker was significantly increased in ASD individuals compared to controls, suggesting that ASD individuals exhibit an abnormality in ASR that is specific to weak auditory stimuli. The current study also investigated the relationships between quantitative autism traits and startle measures. Spearman’s rank order correlations and stepwise multiple regression analyses revealed significant relationships between several autistic traits, responses to weak stimuli and peak startle latency. Specifically, the relationship of PSL and ASR85 to SRS total score and most of the subscales of SRS were significant in both analyses. Our results suggest that prolonged peak startle latency and greater startle response to acoustic stimuli at 85 dB or weaker are atypical in children with ASD and related to autistic traits.

The ASR is a commonly used neurophysiological measure for evaluating various aspects of information processing, which can be examined using similar nonlinguistic experimental paradigms across ethnic groups and species. The ASR is mediated by a relatively simple oligosynaptic pathway comprising the cochlear root neurons, the caudal pontine reticular nucleus and motor neurons, and is modulated by neural circuitry involved with the striatum, hippocampus, thalamus, amygdala, and frontal and parietal cortical regions [9]. The greater startle response to weaker acoustic stimuli and longer startle latency in ASD might be associated with disruption of the basic startle reflex pathway involving these brainstem circuits. Future studies using startle measures of peak startle latency and startle magnitude at acoustic stimuli of 85 dB or weaker might extend translational research into ASD, and elucidate the neural mechanisms underlying ASD.

A major limitation of the current study was the small sample size of ASD individuals. Although we were able to detect significantly prolonged startle latencies and greater startle magnitude in response to weak stimuli in ASD individuals, our sample size may have been insufficient for detecting other significant differences or relationships (type II error). We found no significant gender differences in terms of the relationship between SRS scores and startle measures in the ASD group. However, these findings may have been due to the small size of our ASD group. In addition, none of our subjects exhibited intellectual disability. ASD individuals with intellectual disability might exhibit a different ASR profile. Thus, studies with a larger sample size including subjects with intellectual disability should be conducted in future.

The second limitation is related to our startle paradigm. Several studies have reported atypical pitch processing in individuals with autism [25,26]. Because we employed broadband white noise, it is possible that the startle response to weak stimuli was elicited by exposure to a specific pitch range. In addition, recent studies have reported decreased habituation [27] or increased sensitization [28] of the ASR in people with ASD. Thus, habituation or sensitization to the stimuli may have affected our results. The latter study [28] also reported that the ASR was elicited by less intense prestimuli in people with ASD compared with controls, suggesting that this population may direct more attention to smaller stimuli. Thus, the larger ASR observed in people with ASD in response to weak stimuli might be related to the amount of attention directed towards such stimuli. Future investigations of habituation and sensitization should continue to investigate the role of attention using different pitches of weak acoustic stimuli.

The third limitation is that we only examined average peak startle latencies across all intensities, and did not investigate the peak startle latency for each intensity or the startle onset latency. In this study, we evaluated the peak startle latency of distinct ASR, which was larger than 60 microvolts. However, we were not able to detect such a distinct ASR in response to weak stimuli in some TD subjects. Thus, we averaged the peak startle latency of the ASR across all the startle intensities, and did not average for each stimuli intensity. Because we found significant differences in the ASR produced by weak stimuli, we speculate that the peak startle latency in response to such stimuli might have been even more different between the groups. Additionally, evaluating the startle onset latency may produce more information about atypical auditory processing in ASD. Future studies investigating these factors may shed light on the basis of atypical information processing in people with ASD.

## Conclusions

In conclusion, the current results revealed that greater startle magnitude in response to weak stimuli and prolonged peak startle latency were related to several aspects of ASD characteristics. The current results suggest that a comprehensive investigation of ASR, including startle magnitude to weak stimuli and peak startle latency, might extend understanding of the neurophysiological basis of ASD.

## Abbreviations

ADI-R: Autism Diagnostic Interview-Revised; ADOS: Autism Diagnostic Observation Schedule; ASD: Autism spectrum disorders; ASR: Acoustic startle reflex; DSM-IV-TR: Diagnostic and Statistical Manual of Mental Disorders fourth edition, text revision; IQ: Intelligence quotient; PSL: Peak startle latency; SPL: Sound pressure level; SRS: Social Responsiveness Scale; TD: Typical development; WISC-III: Wechsler Intelligence Scale for Children third edition.

## Competing interests

All authors declare that they have no competing interests.

## Authors’ contributions

HT conceived the study, participated in its design, supervised the entire project, collected the data, performed the statistical analyses and drafted the manuscript. YK also supervised the entire project, confirmed the research diagnoses of ASD and TD, was critically involved in the collection and analysis of the data, and drafted the manuscript. TN, SK, KO and YI were involved in the collection of the majority of the data, and helped to draft the manuscript. All authors read and approved the final manuscript.

## Supplementary Material

Additional file 1 Startle response measurement, including: 1) apparatus and stimuli; 2) stimulus sequence; 3) procedure; and 4) response scoring and data reduction. Click here for file
