# Supplementary material for: Hyperreactivity to weak acoustic stimuli and prolonged acoustic startle latency in children with autism spectrum disorders
Source: Mol Autism. 2014 Mar 12;5:23. doi: 10.1186/2040-2392-5-23 (PMC4008133; doi:10.1186/2040-2392-5-23)
Supplement: Additional file 1 — Startle response measurement, including: 1) apparatus and stimuli; 2) stimulus sequence; 3) procedure; and 4) response scoring and data reduction. [file 2040-2392-5-23-S1.doc]

1. Startle response measurement
   1. Apparatus and stimuli

A commercial computerized human startle response monitoring system (Startle Eyeblink Reflex Analysis System Map1155SYS, NIHONSANTEKU Co., Osaka, Japan) was used to deliver acoustic startle stimuli, and record and score the corresponding electromyographic activity. Stimulus presentation and data acquisition were controlled through a laptop computer with Windows XP operating system installed on it. Sound pressure levels (SPL) were calibrated with precision every 1 dB using Artificial Ears (type 4153, Brüel & Kjaer Sound and Vibration Measurement A/S, Denmark) and a sound level meter NL-20 (RION Co., Kokubunji, Japan). All the auditory stimuli and the background noise were produced by a custom built tone and noise generator and delivered binaurally to the subjects through stereophonic headphones (type DR-531, Elega Acous. Co. Ltd., Tokyo, Japan) with hard plastic bells that were connected to an audio headphone amplifier (type AT-HA20, Audio-Technica Co., Machida, Japan). A 24-bit PCMCIA soundcard (Audigy 2 ZS Notebook, Creative Technologies Ltd., Creative Resource, Singapore) was directly plugged into the stimulus computer.

Startle eyeblink electromyographic responses were recorded from the left orbicularis oculi muscle with a pair of Ag/AgCl disposable electrodes (sensor area 15 mm^2^) filled with wet gel. The first electrode (Blue Sensor N-00-S, Ambu, Ballerup, Denmark) was positioned approximately 1 cm directly below the pupil of the left eye and low enough to not touch the lower eyelid, while the second electrode (Blue Sensor M-00-S, Ambu, Ballerup, Denmark) was placed laterally and slightly superior to the first one, with the centers of the electrodes separated by approximately 2 cm. The impedance between the two electrodes was measured and deemed acceptable if below 10 kΩ. The impedance was measured with an electrode impedance meter (MaP811, NIHONSANTEKU Co., Osaka, Japan) at a measurement frequency of 30 Hz. The ground electrode (Blue Sensor M-00-S) was placed on the left angle of the mandible. The skin area at the electrode site was cleaned with a cotton swab saturated with rubbing alcohol, then prepared by gently rubbing a small amount of NUPREP EEG & ECG Skin Prepping Gel (Bio-Medical Instruments Inc., Warren, USA), and cleaned with a cotton swab saturated with rubbing alcohol again.

Electromyography (EMG) data were measured with an EMG Telemeter (PolyTele EMG, NIHONSANTEKU Co., Osaka, Japan). The measurement condition was adjusted as follows: the time constant was 0.03 s which was equivalent to the low frequency filter of 5 Hz; the high frequency filter was 300 Hz. The sensitivity of the amplifier was 1000 times. The amplification gain control for the EMG signal was kept constant for all subjects. EMG data were digitized with a 12-bit A/D converter (MaP222, NIHONSANTEKU Co., Osaka, Japan) and collected on the PC. The sampling frequency was 1 kHz. Sampling on each trial began 1000 ms prior to the onset of the startle eliciting stimulus and continued for 1000 ms after the onset of the startle eliciting stimulus. The resulting data were baseline corrected with a moving average. The eyeblink magnitude of every startle response was defined as the voltage of the peak activity of the EMG within a latency window of 20–120 ms following startle eliciting stimulus onset. The data were stored and exported for analyses in microvolt values.

- 1. The stimulus sequence

Subjects were tested in a startle paradigm which consisted of three blocks with a continuously presented 60 dB SPL background white noise.

Acoustic stimuli consisted of broadband white noises with an instantaneous rise/fall time lasting for 40 ms presented at intensity from 65 to 110 dB SPL in 5 dB increments. Acoustic stimuli were presented six times at each intensity. All trials were presented in a fixed pseudorandom order, separated by intertrial intervals of 10–20 s (15 s on average). The startle paradigm consisted of a total of 60 trials. The session lasted approximately 20 min, including 5 min acclimation to the background noise.

- 1. Procedure

The experiment took place in a dimly lit electrically and acoustically shielded chamber where the temperature was kept comfortable (approximately 20 degrees Celsius). The EMG recordings were obtained with the subjects sitting comfortably in a chair in a moderately reclined position.

Upon arriving at the laboratory, each subject and their parents read and signed an informed consent form and completed a brief medical history questionnaire including demographic data. The subjects and their parents were informed about the general purpose of the study, about the stimuli and procedure, and that they could withdraw from the study at any time. Subjects and their parents were told that the experiment aimed to measure their reactivity to a number of noise bursts.

Subjects were then seated in the testing room. During the task, subjects watched a silent animated film to minimize boredom and reduce eye movements. Subjects were instructed to pay careful attention to the video on a computer display 70 cm away. Thereafter, the skin area at the electrode site was cleaned and the electrodes were attached. The door to the experimental chamber was closed. Subjects were continuously monitored through a 1-way mirror, and short breaks were offered to ensure alertness and comfort during the recording session.

- 1. Response scoring and data reduction

The following startle response measures were examined: (i) Average startle eyeblink magnitude in ASR to each stimulus intensity, ASR65, ASR70, ASR75, ASR80, ASR85, ASR90, ASR95, ASR100, ASR105, ASR110: Average startle eyeblink magnitude in ASR at stimulus intensities of 65 dB, 70 dB, 75 dB, 80 dB, 85 dB, 90 dB, 95 dB, 100 dB, 105 dB, and 110 dB, respectively; (ii) the average peak startle latency (PSL): Average peak startle latency of ASR among the trials which had ASR larger than 60 microvolts. Prior to data analyses, trials were discarded if the voltage of their peak EMG activity within a latency window of 0–20 ms following startle-eliciting stimulus onset was more than 60 microvolts.
